# Reflexive Laboratory-Based Cryptococcal Antigen Screening and Preemptive Fluconazole Therapy for Cryptococcal Antigenemia in HIV-Infected Individuals With CD4 <100 Cells/µL: A Stepped-Wedge, Cluster-Randomized Trial

**DOI:** 10.1097/QAI.0000000000001894

**Published:** 2018-11-05

**Authors:** David B. Meya, Agnes N. Kiragga, Elizabeth Nalintya, Bozena M. Morawski, Radha Rajasingham, Benjamin J. Park, Anthony Mubiru, Jonathan E. Kaplan, Yukari C. Manabe, David R. Boulware

**Affiliations:** * Department of Research, Infectious Diseases Institute, Makerere University, Kampala, Uganda;; † Division of Infectious Diseases and International Medicine, Department of Medicine, University of Minnesota, Minneapolis, MN;; ‡ Department of Medicine, School of Medicine, College of Health Sciences, Makerere University, Kampala, Uganda;; § Division of Foodborne, Waterborne, and Environmental Diseases, Department of International Infection Control, National Center for Emerging and Zoonotic Infectious Diseases, Centers for Disease Control and Prevention (CDC), Atlanta, GA;; ║ Division of Global HIV and TB, Center for Global Health, CDC, Atlanta, GA; and; ¶ Division of Infectious Diseases, Department of Medicine, Center for Clinical Global Health Education, Johns Hopkins University School of Medicine, Baltimore, MD.

**Keywords:** cryptococcus, HIV, fluconazole, preventative therapy, cryptococcal meningitis, clinical trial

## Abstract

**Background:**

HIV-infected persons with cryptococcal antigenemia (CrAg) are at high risk for meningitis or death. We evaluated the effect of CrAg screening and preemptive fluconazole therapy, adjunctive to antiretroviral therapy (ART), on 6-month survival among persons with advanced HIV/AIDS.

**Methods:**

We enrolled HIV-infected, ART-naive participants with <100 CD4 cells/µL, in a stepped-wedge, cluster-randomized trial from July 2012 to December 2014 at 17 Ugandan clinics. Clinics participated in a prospective observational phase, followed by an interventional phase with laboratory-based, reflexive CrAg screening of residual CD4 count plasma. Asymptomatic CrAg+ participants received preemptive fluconazole therapy. We assessed 6-month survival using Cox-regression, adjusting for nadir CD4, calendar time, and stepped-wedge steps.

**Results:**

We included 1280 observational and 2108 interventional participants, of whom 9.3% (195/2108) were CrAg+. CD4-, time-, and stepped-wedge–adjusted analyses demonstrated no difference in survival in the observational vs the interventional arms (hazard ratio = 1.34; 95% confidence interval: 0.86 to 2.10; *P* = 0.20). Fewer participants initiated ART in the interventional (73%) versus the observational phase (82%, *P* < 0.001). When ART initiation was modeled as a time-dependent covariate or confounder, survival did not differ. However, 6-month mortality of participants with CrAg titers <1:160 and CrAg-negative patients did not differ. Patients with CrAg titers ≥1:160 had 2.6-fold higher 6-month mortality than patients with titers <1:160.

**Conclusions:**

We observed no overall survival benefit of the CrAg screen-and-treat intervention. However, preemptive antifungal therapy for asymptomatic cryptococcosis seemed to be effective in patients with CrAg titer <1:160. A more aggressive approach is required for persons with CrAg titer ≥1:160.

## Abstract 2

Supplemental Digital Content is Available in the Text.

## INTRODUCTION

In sub-Saharan Africa, cryptococcal meningitis has 6-month case fatality rates of >50% in routine care^1,2^ and causes 15% of AIDS-related deaths.^3–5^ The transition from asymptomatic infection to symptomatic cryptococcosis occurs over weeks to months.^6^ During this period, cryptococcal antigen (CrAg) is detectable in blood and is an independent predictor of meningitis or death.^6–8^ Approximately 20%–25% of HIV-infected patients in sub-Saharan Africa present with CD4 counts <100 cells/μL.^7,9–12^ The average prevalence of cryptococcal antigenemia among this population is 5%–9%.^13,14^ Therefore, strategies to reduce potentially treatable opportunistic infection-related deaths among persons presenting to HIV care with low CD4 counts, in addition to initiation of antiretroviral therapy (ART), remain relevant. One such strategy is to screen for and preemptively treat asymptomatic, disseminated cryptococcal infection.^8,15^

Current World Health Organization (WHO) guidelines recommend (1) pre-ART CrAg screening for HIV-infected patients with <100 CD4 cells/µL, and (2) for CrAg-positive (CrAg+) persons, administration of preemptive antifungal therapy using fluconazole 800 mg daily for 2 weeks followed by 400 mg daily for 8 weeks, and then maintenance therapy with fluconazole 200 mg daily. In addition, the current WHO guidelines recommend lumbar puncture with cerebrospinal fluid (CSF) examination to exclude meningitis among asymptomatic CrAg+ patients where feasible.^16^ A randomized controlled trial among ART-naive persons with <200 CD4 cells/μL found that mortality was 28% [95% confidence interval (CI): 10% to 43%] lower among persons receiving CrAg screening and 4 ART adherence support home visits compared to those receiving standard of care.^17^ The attributable benefit of each of these interventions (CrAg screening vs home visits) could not be determined.

We designed the Operational Research for Cryptococcal Antigen Screening (ORCAS) trial as a stepped-wedge, cluster-randomized trial to evaluate the survival benefit of CrAg screening and preemptive treatment of asymptomatic cryptococcal antigenemia, as an adjunct to ART, on a population level among HIV-infected persons with CD4 <100 cells/μL. We hypothesized that CrAg screening and preemptive treatment of asymptomatic CrAg+ persons with short-course, high-dose fluconazole would improve survival and reduce the incidence of symptomatic cryptococcal disease.

## METHODS

### Study Population and Setting

We screened HIV-infected patients with a CD4 <100 cells/µL at 17 outpatient HIV clinic sites, including 11 urban and 6 rural sites in Uganda from July 2012 through December 2014. The rural district clinics were located up to 8 hours (530 km) driving distance from Kampala. Inclusion criteria for participation were age ≥14 years, HIV infection, ART-naive, and CD4 count <100 cells/µL. Patients were excluded from the study and referred for therapy if they exhibited symptoms of meningitis.

### Study Design and Randomization

The project had 2 components. The first was a cluster-randomized trial in which the screen-and-treat intervention was initiated at the Infectious Diseases Institute Clinic in Kampala, followed by initiation in a randomly selected cluster of 2 Kampala Capital City Authority clinics every 2 months. Thus, a total of 9 clinics were included in the randomized, stepped-wedge design, although only 8 included both an interventional and an observational arm. The stepped-wedge design was chosen to accommodate the on-going adoption of WHO guidelines and the likely roll-out of CrAg screening in Uganda when the trial was initiated, and to enable staggered training of clinical staff, laboratory personnel, and pharmacists. Each cluster had an initial observational phase in which patients initiating ART received a clinical meningitis symptom screen but no CrAg screening. This was followed by an interventional phase in each clinic, which included CrAg screening and initiation of fluconazole preemptive therapy for CrAg+ persons before initiation of ART.

The second component of this trial included a nonrandomized cohort in which CrAg screening was expeditiously rolled out in 2 additional urban sites (the AIDS Support Organization clinic and Kasangati Health Center), and 6 rural HIV clinics in Kiboga, Kagadi, Kikuube, Fort Portal, Koboko, and Magale. Each of these sites also included an observational phase before the intervention phase.

### Observational Phase

A study nurse was dedicated to identifying new ART-naive patients with <100 CD4 cells/µL from laboratory records. CD4 testing was performed at the Makerere University-Johns Hopkins laboratory with some clinics additionally performing CD4 tests using the point-of-care PIMA instrument (Alere, Waltham, MA). At the patient's initial clinic visit, eligibility criteria for enrollment were confirmed. Participants were subsequently followed through monthly HIV clinic visits, during which nurses collected data on incident opportunistic infections, ART initiation and regimen, and 6-month outcome through medical records. Patients who did not return within 2 weeks of their scheduled appointment were contacted. Patients who never returned for their CD4 result or who missed 3 consecutive monthly visits were classified as lost to follow-up. Any patient who was lost to follow-up was traced via home visit and/or phone call(s) to ascertain their vital status.^18^

### Interventional Phase

During the interventional phase, routine clinic activities continued as above. In addition, CrAg testing was reflexively performed on residual plasma from patients with <100 CD4 cells/µL via lateral flow assay (Immy, Norman, OK).^19^ Patients with a positive CrAg were contacted by telephone after the test result and asked to return to clinic within 48 hours.

Study nurses further assessed asymptomatic CrAg+ participants for eligibility for the preemptive fluconazole intervention. Exclusion criteria for receipt of preemptive fluconazole included suspected meningitis, cryptococcal meningitis history, or fluconazole contraindications. CrAg+ participants were evaluated by a physician and referred for a lumbar puncture if there was clinical suspicion of meningitis (eg, headache, photophobia, and neck pain). Patients with CrAg+ CSF received amphotericin treatment^20^ and were excluded from the analysis of preemptive fluconazole therapy. Participants without clinical suspicion of meningitis did not have lumbar punctures performed.

Asymptomatic CrAg+ participants received fluconazole 800 mg daily for 2 weeks followed by 400 mg daily for 8 weeks.^20^ (WHO guidelines recommending 200 mg of daily fluconazole after 10 weeks were not issued until after completion of this study.) ART initiation was scheduled for 2 weeks after fluconazole initiation, and clinic visits occurred monthly thereafter during the 6-month follow-up period. Adherence to fluconazole was determined by self-report and pill counts when participants returned for follow-up visits 2, 6, and 10 weeks after initiating fluconazole.

CrAg-negative participants initiated ART approximately 2 weeks after their CD4 blood draw and were followed monthly per standard clinic protocol. Study nurses followed all participants with <100 CD4 cells/μL, tracked those who were lost to follow-up and reviewed their chart during follow-up.

During follow-up, participants diagnosed with cryptococcal meningitis were managed per existing national guidelines, which included amphotericin (1 mg/kg) in combination with fluconazole 800 mg for 2 weeks, 2–3 therapeutic lumbar punctures weekly, potassium and magnesium presupplementation, pain management, creatinine and electrolyte monitoring, and preamphotericin hydration with a liter of normal saline daily.

Institutional review boards at the Joint Clinical Research Centre in Uganda, Johns Hopkins University, and University of Minnesota approved the trial. The trial was registered with the Uganda National Council of Science and Technology (#HS 1254) and ClinicalTrials.gov (NCT01535469). An independent Data Safety and Monitoring Board (DSMB) reviewed the interim analyses annually.

### Study Analyses

The trial included 2 coprimary analyses. The first was a comparison of 6-month survival in the observational vs the interventional phases. This analysis initially compared survival in the observational phase with survival in the interventional phase among persons enrolled in the 9 clinics during the stepped-wedge, cluster-randomized trial. At the time of the second interim analysis, the DSMB recommended that CrAg screening be rolled out expeditiously at 8 additional clinics, which were to be randomized in the second year of the trial, following adoption of the CrAg screening intervention in the Uganda national guidelines. Our final analysis included participants in all 17 study sites. The second coprimary analysis, limited to the interventional phase, compared survival between CrAg+ and CrAg-negative participants from all 17 clinics.

The incidence of symptomatic central nervous system disease, all-cause early fluconazole discontinuation, and serious adverse events per the 2009 Division of AIDS toxicity scale were assessed as secondary endpoints.^21^ A panel of 3 investigators, not blinded to randomization cluster or managed participant care, adjudicated cause of death (D.B.M., Y.C.M., and D.R.B.) in the 34 CrAg+ participants who died.

### Statistical Analysis: Survival in the Observational vs the Interventional Arm

We compared the primary endpoint of 6-month survival between the observational and interventional phases using Cox proportional hazards models, adjusting for nadir CD4 count at screening, calendar time, and the steps of intervention roll-out. Standard errors were adjusted for within-randomized cluster correlation. The trial was powered to detect a hazard ratio (HR) of ≥1.15 for 6-month survival with 80% power and an overall one-sided alpha level of 0.05 for superiority for the interventional vs the observational phase, with an intended sample size of 2190 participants.

Analysis was first performed per the intention-to-treat principle among all adults with a CD4 measurement of <100 cells/µL at the 17 clinics. Time-at-risk was calculated as the date of CD4 count to date of death or last contact. Because of the strong effect of ART initiation on mortality, and the observation that ART initiation was differentially distributed between the study phases (see below), a posteriori subanalyses of ART initiators among participants who returned to clinic and initiated ART during the 6-month follow-up period were conducted using Cox proportional hazards models, adjusted for time to ART initiation from CD4 blood draw, nadir CD4, and randomization steps. We also clustered SEs around clinic, to account for within-clinic correlation. Time-at-risk was calculated as the date of ART initiation to date of death or last date of contact. We also performed 2 additional analyses to address the differential distribution of ART start between the study arms: in the first, ART initiation was modeled as a time-dependent covariate. In the second, differential ART initiation was treated as a form of confounding by indication (bias); a counterfactual model was used where we modeled the average time to mortality if all participants initiated ART in both study phases using a survival treatment effects model with inverse probability weighting.

### Statistical Analysis: Survival in CrAg+ vs CrAg-Negatives in the Interventional Arm

We also compared patients in the interventional phase with known CrAg status by evaluating the differences in all-cause mortality between participants who were CrAg-negative, CrAg+ with titers <1:160, and CrAg+ with titers ≥1:160. Although not part of the initial study design, this CrAg titer threshold was chosen based on a post hoc survival analysis among CrAg+ participants, using exploratory CrAg titer cutoffs to evaluate all-cause mortality and incident meningitis or death within 6 months as a composite outcome; the results showed a higher risk among those with CrAg+ titers ≥1:160.

We used a log-rank test to assess differences in Kaplan–Meier survival curves across these groups. We also assessed risk factors for preemptive treatment failure. Fluconazole resistance was also evaluated using broth microdilution among patients who failed fluconazole therapy and developed cryptococcal meningitis.

For all analyses, missing dates of death among participants who were known to be deceased were imputed by randomly generating a date within 14 days from their last date of contact, either date of CD4 blood draw, first clinic visit, or ART initiation. Patients who never returned to clinic after their CD4 blood draw and were lost to follow-up with an unknown outcome even after tracing were randomly assigned a censoring date between 1 and 14 days of their CD4 blood draw date. All analyses were conducted using Stata/SE 12.1 (StataCorp, College Station, TX), and results were evaluated against a 2-sided type I error rate of 0.05.

## RESULTS

**FIGURE 1.**
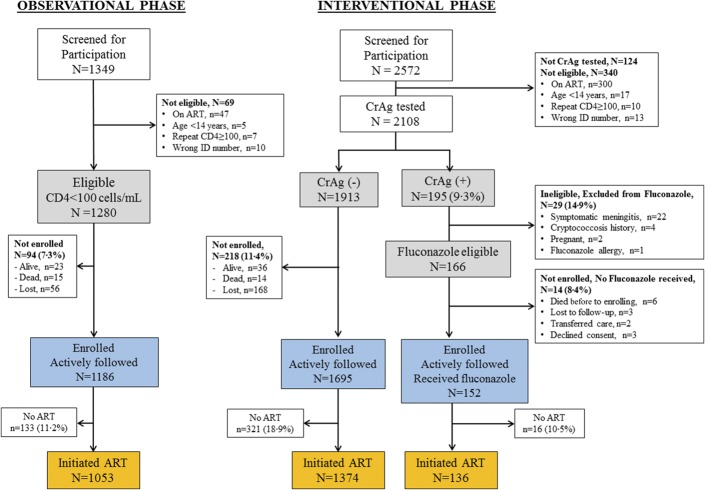
Consort diagram: Patients on ART with a CD4 <100 cells/µL were excluded during screening. Similarly, we excluded CrAg-positive persons who were seen in the clinic by the nurse counselor after having already initiated ART (n = 18). 4.8% (124 of 2572) did not have a CrAg test performed due to insufficient amount of plasma, or the leftover plasma was inadvertently discarded before CrAg testing. CrAg (−), cryptococcal antigen negative; CrAg (+), cryptococcal antigen positive.

During the observational and the interventional phases, 1349 patients and 2572 patients were screened for participation, respectively. We enrolled and followed 1280 participants with <100 CD4 cells/µL during the observational phase and 2108 eligible participants with <100 CD4 cells/µL during the interventional phase (Table 1 and Fig. 1). The primary reason for ineligibility was already receiving ART. Median age, median CD4, and gender distribution were similar in the observational and interventional phases (Table 1). Among those who started ART, the median time from CD4 draw to ART initiation decreased from 34 [interquartile range (IQR), 21–49] days during the observational phase to 28 (IQR, 17–42) days during the interventional phase (*P* < 0.001). However, during the interventional phase, fewer persons initiated ART (73% in the interventional phase compared with 82% in the observational phase, *P* < 0.001).

**TABLE 1.**
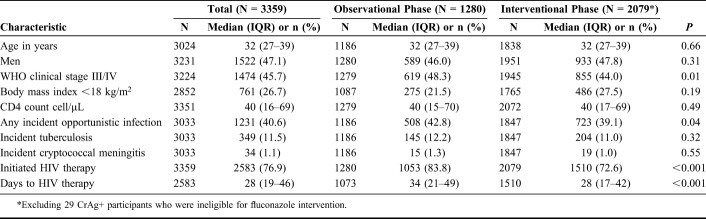
Demographic and Clinical Characteristics of Participants Included in the Primary Analysis of the CrAg Screening Intervention

### Comparison of Survival in the Observational vs the Interventional Arm

**FIGURE 2.**
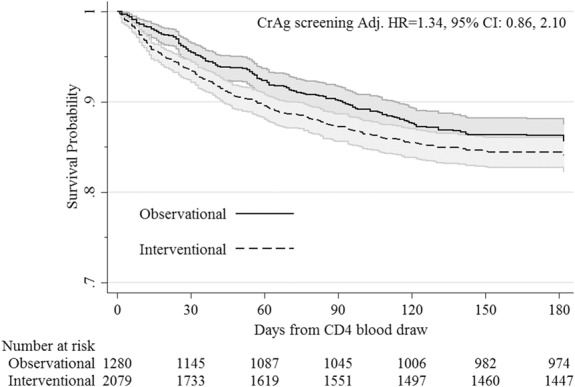
Survival in the observational vs interventional CrAg screening phase among ART-naive patients with CD4 <100 cells/µL and were otherwise eligible for the intervention. HR adjusted for CD4 count, stepped-wedge step, and year of screening, and accounts for within-cluster correlation. Twenty-nine CrAg+ persons were excluded due to fluconazole intervention ineligibility or declined consent. CrAg, cryptococcal antigen.

We found 24.8% (317/1280) of participants in the observational phase died by 6 months, compared with 30.4% (632/2079) in the interventional phase. Per intention-to-treat analysis, survival did not differ between the 2 phases among eligible participants in nadir CD4-, time-, and wedge step–adjusted analyses (HR = 1.34; 95% CI: 0.86 to 2.10; *P* = 0.20; Fig. 2).

Because of the unexpected decrease in the percentage of persons who initiated ART in the interventional phase, we repeated this analysis among participants who returned to initiate ART during the study period (Fig. 1, orange boxes); the CrAg screening intervention did not improve survival in the interventional phase (HR for survival in the observational vs the interventional phase = 1.11; 95% CI: 0.62 to 1.79; *P* = 0.86), after adjusting for baseline CD4 count, wedge step, calendar time, time to ART initiation, and accounting for within-cluster correlation. We also performed 2 additional analyses: in the first, ART initiation was modeled as a time-dependent covariate; and in the second, differential ART initiation was treated as a form of confounding by indication (bias). In both of these additional analyses, survival did not differ between the interventional and the observational arms.

### Comparison of Survival in CrAg+ vs CrAg− Persons in the Interventional Phase

During the interventional phase, of the 2448 patients who received reflexive CrAg testing, we identified 14% (340/2448) who were not eligible or unable to be enrolled in the trial (Fig. 1). Thus, 2108 total patients met screening eligibility criteria. All eligible, CrAg-screened participants were included in the prospective cohort, evaluating outcomes among asymptomatic CrAg+ vs CrAg-negative participants.

Of the 2108 persons who were eligible for screening and who had a CrAg test performed, 9.3% (195/2108) were CrAg+ (Fig. 1). Of these 195 CrAg+ persons, 29 were not eligible for preemptive fluconazole (Fig. 1). Twenty-two had symptoms of meningitis, and 2 were pregnant. Of the 166 CrAg+ participants eligible for preemptive treatment, 9 died or were lost to follow-up, 2 transferred to another ART clinic, and 3 declined consent. Thus, among the CrAg+ persons, 152/195 (78%) were eligible for the preemptive fluconazole intervention and initiated fluconazole at a median 7 days (IQR, 3–12, maximum of 66 days) after the CD4 blood draw.

Mean adherence to fluconazole was 92% of expected doses. ART was initiated by 89% (136/152) of enrolled CrAg+ participants a median of 14 days (IQR, 14–15) after receiving fluconazole and a median of 22 days (IQR, 18–28) after CD4 blood draw. Among the eligible CrAg-negative patients, only 72% (1374/1913) initiated ART within the first 6 months of their <100 CD4 cells/µL result. Among those who started ART, the median time to ART initiation among CrAg-negative participants was 28 days from CD4 blood draw (IQR, 16–44 days).

Among the 152 asymptomatic CrAg+ participants who received preemptive fluconazole, 6-month survival was 79.6% (95% CI: 72 to 85). Among eligible CrAg-negative participants, 6-month survival was 84.7% (95% CI: 83 to 86; log rank *P* = 0.07). In total, 7.9% (12/152) CrAg+ failed their preemptive fluconazole therapy and developed overt cryptococcal meningitis within 6 months of their CD4 blood draw. Among patients with 6-month breakthrough meningitis, 83% (10/12) died before 6 months of follow-up. Three further participants had meningitis with CSF CrAg-negativity and died.

Fluconazole susceptibility testing among 8 participants with breakthrough cryptococcal meningitis showed that 7 of 7 participants with available data had fluconazole resistance (minimum inhibitory concentration >64 μg/mL) (see Table 1, Supplemental Digital Content, http://links.lww.com/QAI/B233). Adverse events among patients taking fluconazole were minimal with elevated alanine aminotransferase (>3 times the upper limit of normal) in 2% (3/152) of participants.

**FIGURE 3.**
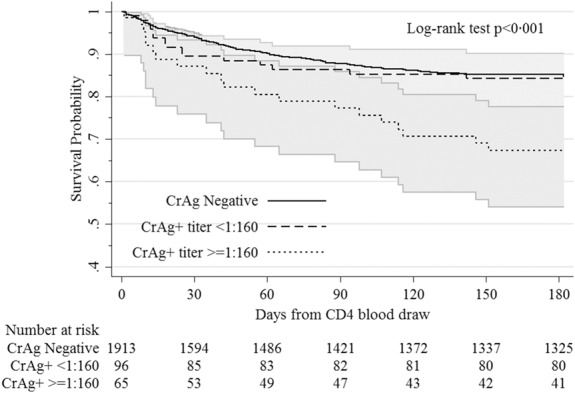
Survival with CrAg screening intervention among HIV-infected adults with CD4 <100 cells/µL by CrAg status. CrAg (−), cryptococcal antigen negative; CrAg (+), cryptococcal antigen positive.

**FIGURE 4.**
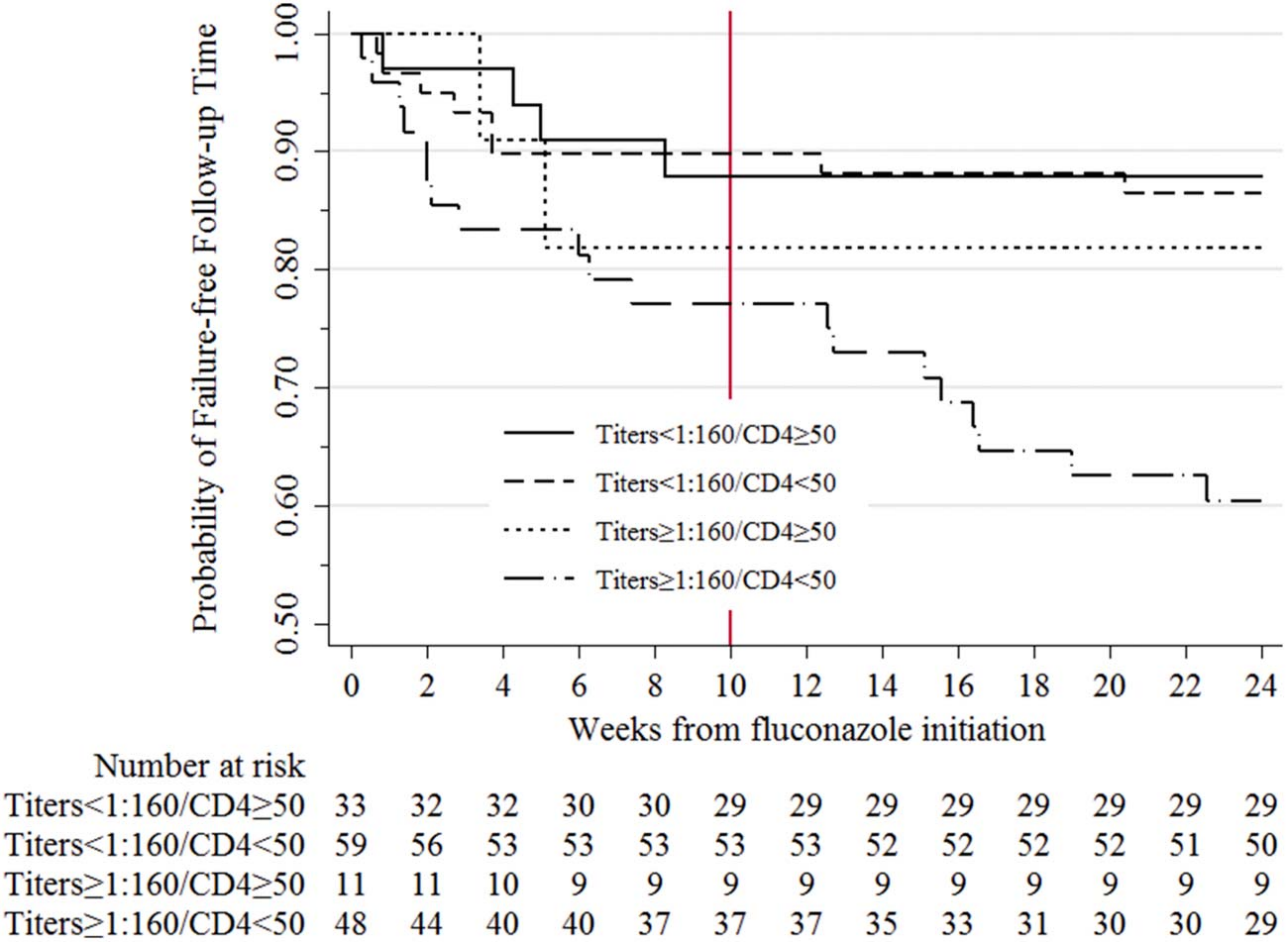
Incident meningitis or death among patients receiving preemptive fluconazole therapy stratified by baseline plasma CrAg titer and CD4. One participant did not have a baseline CrAg titer measured due to insufficient volume of plasma. Gray line indicates end of fluconazole therapy.

Among the 151 enrolled CrAg+ participants with baseline titers performed, 39% (59/151) had a CrAg titer of ≥1:160. As shown in Figure 3, CrAg-negative and <1:160 titer CrAg+ participants demonstrated similar 6-month mortality. Survival probability among CrAg-negative participants was 0.85 (95% CI: 0.83 to 0.86) vs 0.84 (95% CI: 0.75 to 0.90) among CrAg+ participants with <1:160 titers. Among CrAg+ participants with ≥1:160 titers, 6-month survival probability was 0.67 (95% CI: 0.54 to 0.78; log-rank comparing all groups <0.001). Of the CrAg+ participants with baseline plasma CrAg titer of ≥1:160, 36% (21/59) failed preemptive fluconazole therapy and either developed meningitis or died despite receiving preemptive fluconazole therapy compared with 13% (12/92) among participants with CrAg titer <1:160 (Fig. 4; HR = 2.6; 95% CI: 1.2 to 5.3; *P* = 0.01). On further analysis, considering the interaction between CD4 count and CrAg titers, those with CD4 <50 cells/µL and titer ≥1:160 were at higher risk of meningitis or death (HR = 3.3; 95% CI: 1.1 to 9.8; *P* = 0.03) compared with those who had a CD4 >50 cells/µL and CrAg titer <1:160 (Fig. 4). Because of the higher rate of ART initiation in the CrAg+ vs the CrAg-negative participants, we repeated the comparisons of survival, treating ART initiation as a time-varying covariate. The results showed no difference in mortality between the CrAg+ persons with titer <1:160 vs CrAg-negative persons (HR 1.00, 95% CI: 0.42 to 2.39, *P* = 0.994) but higher mortality among CrAg+ persons with titer ≥1:160 vs CrAg-negative persons (HR = 2.10, 95% CI: 1.30 to 3.38, *P* = 0.002).

## DISCUSSION

In our cluster-randomized trial, we did not demonstrate that laboratory-based reflexive CrAg screening with fluconazole preemptive therapy increased 6-month survival among persons with <100 CD4 cells/µL. To date, there have been no studies evaluating the survival impact of CrAg screening at the clinic population level as an isolated intervention. Previously, a randomized controlled trial demonstrated that CrAg screening when combined with ART, weekly community adherence support visits for the first month of ART initiation, and tuberculosis rescreening 2 months after ART initiation conferred a 28% survival benefit through 12 months in Tanzania and Zambia.^17^ However, this study was unable to differentiate the effect of CrAg screening from the other interventions that were included. Given the well-demonstrated survival benefit of ART in persons with advanced HIV,^22^ if CrAg screening is implemented as part of the package of care for advanced HIV disease, it should not be a hindrance to starting ART.

Among persons with advanced HIV in whom CrAg testing had been performed, persons with CrAg titer <1:160 who received preemptive therapy had similar outcomes as CrAg-negative persons with CD4 <100. Previous studies demonstrate mortality among CrAg+ individuals of >50%.^8,15,23^ Our data suggest that among CrAg+ individuals with low CrAg titer (<1:160), reflexive laboratory-based CrAg screening and preemptive fluconazole therapy mitigates CrAg-related mortality. However, for CrAg+ persons with titers ≥1:160, preemptive therapy of 800 mg daily for 2 weeks followed by 400 mg daily for 8 weeks was insufficient. Still, 64% of individuals with CrAg titers ≥1:160 survived—better than the historical experience of near-zero survival without fluconazole therapy.^8,15^ Taken together, these observations strongly support adjunctive therapy in asymptomatic persons with advanced HIV who are discovered to be CrAg+ at the time they initiate ART, although differentiated care strategies may be warranted depending on antigen titer. Semiquantitative CrAg lateral flow assays are currently under development by commercial companies and may be available as early as 2019.

We found that the effectiveness of the 10-week fluconazole preemptive therapy regimen was dependent on the initial CrAg titer and possibly fluconazole-resistant strains. A baseline plasma CrAg titer of ≥1:160 was associated with higher failure of preemptive therapy, as reported in smaller studies.^8,24,25^ This mortality risk among CrAg+ persons was further amplified when CD4 counts were <50 cells/µL, with 42% mortality among those with low CD4 and titer ≥1:160. We did not perform lumbar punctures in these asymptomatic patients at baseline—an approach that was agreed on in advance to make the study applicable to busy health care settings in sub-Saharan Africa. Therefore, it is possible that some CrAg+ persons had central nervous system infection at baseline. Patients with concerning symptoms did have lumbar punctures to exclude meningitis. Our findings suggest that, if possible, a lumbar puncture should be performed in asymptomatic CrAg-positive persons, especially in those with baseline CrAg titers ≥1:160.^24^

Our study was limited by the rapid expansion of ART access and a lower proportion of participants starting ART in the intervention arm. It is unlikely that the reflexive CrAg screening, which occurred unbeknownst to patients, was related to lower return rates to clinic for CD4 results across arms. Rather, the differential return and ART initiation rate likely resulted from the large increases in the number of patients enrolled in care during ART expansion with limited staffing in the clinics. In addition, the study nurse counselors focused on the CrAg+ participants to treat them promptly and may have spent less time focusing on the CrAg-negative participants. This observation, which was unexpected, required us to reassess our findings in light of this potential bias toward improved outcomes in the interventional arm. However, analysis limited to persons who started ART, as well as 2 additional analyses addressing the differential rates of ART initiation, similarly showed no survival benefit of the screen-and treat-intervention. Although the cluster randomization design may have posed a limitation in ascertaining the effectiveness of the intervention at the individual level, the design was suitable for understanding the effectiveness of the intervention at the population level.

Although, in the intervention phase of the study, fluconazole was initiated at median of only 7 days after report of the CD4 count in CrAg+ persons, another potential limitation of the study was the longer delay in therapy for some CrAg+ individuals (range up to 66 days). Such a delay could impact the effectiveness of the intervention. As rapid ART initiation has been found to be feasible and advantageous,^26,27^ finding a way to incorporate CD4 and CrAg screening into the initial intake rather than as a reflexive laboratory-based testing may be important in maximizing individual patient benefit without compromising overall outcomes of late presenters who urgently need ART, which should not be delayed.

Other limitations of this study include the absence of definite causes of death and difficulty in assessing endpoints—particularly among CrAg-negative or unknown status patients. In the observational phase, when routine CrAg screening was not performed, very few cases of cryptococcal meningitis were identified clinically. Once laboratory CrAg results were available to clinicians, 1% (22/2140) of persons were identified with symptomatic cryptococcal meningitis at clinic entry compared with zero in the observational phase.

In conclusion, we failed to observe a survival benefit of laboratory-based reflexive CrAg screening and preemptive fluconazole treatment among all patients with <100 CD4 cells/µL. The currently recommended preemptive therapy might be satisfactory for persons with low CrAg titers of <1:160 but is not optimal in patients with higher CrAg titers ≥1:160.^16^ Given the strong survival benefit of ART, these results support that, if the decision is made to implement the intervention, CrAg screening and preemptive treatment should be implemented in such a way as not to interfere with timely initiation of ART in patients with advanced HIV disease. Effective treatment of asymptomatic cryptococcal antigenemia in persons with CrAg titers of ≥1:160 warrants further investigation.

## Supplementary Material

SUPPLEMENTARY MATERIAL

## Appendix 1. ORCAS Team Members

Robert Mutumba, Henry W. Nabeta, Tibakabikoba Harriet, Immaculate Nabwire, Mugara Kabahweza Hellen, Kabahubya Mable, Jalia Birabwa, Rachel Nanono, Sylvia Namanda, Rose Naluyima, Boniface Satya, Florence Kugonza, Grace Banatema, Sarah Coutinho, Susan Naikoba, Grace Menya, Fred Turya, Justine Mbambu, Salome Athieno, Josephine Badaru, Lydia Kabiri, Robert Bbosa, Tif Agaba, Bbosa Rwesiba Bernard, Joyce Nguna, Francis Kakooza, Andrew Akampurira, Denis Wamai, Jane Akello, Michael Enyakoit, and Catherine Nanteza.
