# Supplementary material for: Reflexive Laboratory-Based Cryptococcal Antigen Screening and Preemptive Fluconazole Therapy for Cryptococcal Antigenemia in HIV-Infected Individuals With CD4 <100 Cells/µL: A Stepped-Wedge, Cluster-Randomized Trial
Source: J Acquir Immune Defic Syndr. 2018 Nov 5;80(2):182–9. doi: 10.1097/QAI.0000000000001894 (PMC6339522; doi:10.1097/QAI.0000000000001894)
Supplement: SUPPLEMENTARY MATERIAL [file qai-80-182-s001.docx]

**Supplemental Table 1: Characteristics of enrolled patients with breakthrough cryptococcal meningitis**

| ID | Screening CrAg Titer | Week 6 CrAg Titer | Week 26 CrAg Titer | Days to Fluconazole | Days to ART | Days to cryptococcal meningitis | Fluconazole adherence | ART Adherence | Baseline Symptoms | CSF Culture (CFU/mL) | 26-week Outcome | MIC  (μg/ml) |
| --- | --- | --- | --- | --- | --- | --- | --- | --- | --- | --- | --- | --- |
| 1 | 1:80 | 1:1280 | 1:20 | 4 | 20 | >182 | 100% | 100% | None | LP done by outside hospital | Alive | - |
| 2 | 1:80 | 1:1280 | 1:5120 | 5 | 19 | >182 | 99% | 90% | None | 24,100 | Alive |  |
| 3 | 1:1280 | Missed visit | -- | 4 | 25 | 116 | <50% | 75% | None | 253,000 | Dead | 256 |
| 4 | 1:163840 | N/A | -- | 7 | N/A | 10 | 100% | N/A | None | 108,000 | Dead | 512 |
| 5 | 1:640 | 1:640 | -- | 1 | 15 | 105 | 100% | 100% | None | 127,000 | Dead | 512 |
| 6 | 1:10 | N/A | -- | 3 | N/A | 8 | 100% | N/A | None | Negative | Dead | 128 |
| 7 | 1:40960 | N/A | -- | 5 | N/A | 19 | 100% | N/A | Mild headache | 70,000 | Dead |  |
| 8 | 1:32780 | N/A | -- | 2 | N/A | 1 | 1 day | N/A | None | Died before LP | Dead |  |
| 9 | 1:640 | N/A | -- | 8 | 22 | 28 | 100% | N/A | Mild headache | 200 | Dead | 128 |
| 10 | 1:1280 | 1:1280 | N/A | 12 | 24 | 148 | Missed 8 doses at week 2 | 100% | None | 9,800 | Alive | 512 |
| 11 | 1:1280 | 1:80 | N/A | 5 | 19 | 111 | Missed 14 pills | 90% | None | Negative | Alive |  |
| 12 | 1:2560 | 1:5120 | -- | 0 | 14 | 98 | 100% | 90% | Headache | CSF CrAg positive | Dead | 512 |
| 13 | 1:80 | -- | -- | 13 | Died pre-ART | 18 | 5% | N/A | None | CSF CrAg positive | Dead |  |
| 14 | 1:80 | -- | -- | 0 | N/A | 19 | 92% | N/A | N/A | Clinical diagnosis | Dead | - |

CFU- Colony Forming units; CrAg- Cryptococcal Antigen; ART- Antiretroviral Therapy; CM- Cryptococcal meningitis; MIC- Minimum Inhibitory Concentration; N/A- Result not available
